# Protective Effects of Gemigliptin, a Dipeptidyl Peptidase-4 Inhibitor, against Cisplatin-Induced Nephrotoxicity in Mice

**DOI:** 10.1155/2017/4139439

**Published:** 2017-11-28

**Authors:** Seung Hee Choi, Jaechan Leem, In-Kyu Lee

**Affiliations:** ^1^Department of Biomedical Science, Graduate School, Kyungpook National University, Daegu 41944, Republic of Korea; ^2^Department of Immunology, School of Medicine, Catholic University of Daegu, Daegu 42472, Republic of Korea; ^3^Division of Endocrinology and Metabolism, Department of Internal Medicine, School of Medicine, Kyungpook National University, Daegu 41944, Republic of Korea; ^4^Leading-Edge Research Center for Drug Discovery and Development for Diabetes and Metabolic Disease, Kyungpook National University Hospital, Daegu 41944, Republic of Korea

## Abstract

Dipeptidyl peptidase-4 (DPP-4) inhibitors are widely used antihyperglycemic agents for the treatment of type 2 diabetes mellitus. Recently, the pleiotropic actions of DPP-4 inhibitors have drawn much attention. In the present study, we aimed to examine whether gemigliptin, a recently developed DPP-4 inhibitor, could protect against cisplatin-induced nephrotoxicity. We showed that pretreatment with gemigliptin attenuated cisplatin-induced renal dysfunction, as shown by analysis of plasma creatinine levels and blood urea nitrogen and histological damage. Elevated plasma levels of active glucagon-like peptide-1 were observed in gemigliptin-pretreated mice after cisplatin treatment, compared to that in cisplatin alone-treated mice. Gemigliptin attenuated cisplatin-induced apoptotic cell death, as assessed by terminal deoxynucleotidyl transferase-mediated dUTP nick end labeling and Western blot analysis in the kidneys. Gemigliptin also decreased the plasma levels of tumor necrosis factor-*α* and monocyte chemoattractant protein-1 and attenuated nuclear staining of nuclear factor kappa-B p65 in the kidneys. In addition, gemigliptin increased the protein expression of heme oxygenase-1 (HO-1) and NAD(P)H:quinone oxidoreductase 1 (NQO1) in the kidneys of cisplatin-treated mice. Taken together, these results suggest that pretreatment with gemigliptin protects against cisplatin-induced nephrotoxicity in mice, possibly via inhibition of apoptotic cell death and inflammatory responses through induction of HO-1 and NQO1 expression.

## 1. Introduction

Cisplatin is one of the most widely used chemotherapeutic agents for the treatment of various solid tumors, including testicular, ovarian, cervical, and non-small-cell lung cancer [1]. However, the use of high-dose cisplatin is limited because of its serious side effects, particularly nephrotoxicity. Although the exact mechanisms underlying cisplatin-induced nephrotoxicity remain incompletely understood, it has been suggested that renal tubular cell apoptosis and inflammatory responses play an important role in the pathogenesis of cisplatin-induced nephrotoxicity [2–4].

Dipeptidyl peptidase-4 (DPP-4) inhibitors are effective and safe oral antihyperglycemic agents for the treatment of type 2 diabetes mellitus (T2DM). DPP-4 is an enzyme responsible for the degradation of incretin hormones, including glucagon-like peptide 1 (GLP-1), which enhances postprandial insulin secretion from pancreatic *β*-cells [5]. Inhibition of DPP-4 activity by DPP-4 inhibitors prevents the degradation of GLP-1 in the peripheral circulation, which, in turn, increases the levels of active GLP-1 and reduces the elevated blood glucose levels. Thus, the beneficial effects of DPP-4 inhibitors are believed to be largely mediated by the physiological elevation of endogenous GLP-1 levels and its insulinotropic action on pancreatic *β*-cells. However, the GLP-1 receptor is also widely expressed in extrapancreatic tissues [6], and DPP-4 has several nonincretin substrates, such as cytokines, chemokines, and neuropeptides [5]. In addition to the glucose-lowering actions of DPP-4 inhibitors, emerging evidence suggests that they exhibit pleiotropic extrapancreatic functions, including cardiovascular protective [5, 6], hepatoprotective [7], and renoprotective effects [8]. However, the effects of DPP-4 inhibitors against cisplatin-induced nephrotoxicity and the underlying mechanisms have not been fully elucidated yet. In the present study, we investigated the protective effects of gemigliptin, a potent and highly selective DPP-4 inhibitor [9], against cisplatin-induced nephrotoxicity and explored its underlying mechanisms.

## 2. Materials and Methods

### 2.1. Animal Experiments

To evaluate the effects of gemigliptin (LG Life Sciences Ltd., Seoul, Republic of Korea) on cisplatin-induced nephrotoxicity, 8-week-old male C57BL/6 mice (Central Lab Animal Inc., Seoul, Republic of Korea) were randomly divided into three groups as follows: control (Con, *n* = 6), cisplatin alone (CP, *n* = 6), and cisplatin plus gemigliptin (CP + G, *n* = 6). Mice in the CP and CP + G groups were fed a chow diet and chow diet mixed with gemigliptin (100 mg/kg/day) for 4 days prior to and 3 days after cisplatin treatment, respectively. A single intraperitoneal injection of cisplatin (20 mg/kg; Sigma-Aldrich, St. Louis, MO, USA) in 0.9% normal saline was administered to the mice in the CP and CP + G groups, whereas mice in the Con group received an equivalent amount of normal saline. The dose of gemigliptin was determined based on the results of previous studies [10, 11]. Mice were sacrificed 3 days after cisplatin injection, and blood and kidney tissue samples were collected. Mice were housed at ambient temperature (20–22°C) under a 12 h : 12 h light-dark cycle with free access to water and food. All experimental procedures were performed in accordance with the guidelines for the care and use of laboratory animals of the National Institute of Health (USA) and were approved by the Kyungpook National University Institutional Animal Care and Use Committee.

### 2.2. Plasma Biochemical Assays

Plasma levels of creatinine and blood urea nitrogen (BUN) were measured using an automatic analyzer 7020 (Hitachi, Osaka, Japan). Active GLP-1 plasma levels were determined using an ELISA kit (BioVendor, Brno, Czech Republic), in accordance with the manufacturer's instructions. In addition, plasma levels of tumor necrosis factor-alpha (TNF-*α*) and monocyte chemoattractant protein-1 (MCP-1) were measured using ELISA kits (Merck Millipore, Billerica, MA, USA), according to the manufacturer's instructions.

### 2.3. Renal Histology

The kidneys were rapidly removed from each mouse. The tissues were immediately fixed in 4% paraformaldehyde and embedded in paraffin. Serial (4 *μ*m thick) sections were deparaffinized in xylene, rehydrated using descending grades of ethanol, and stained with hematoxylin and eosin (H&E) and periodic acid-Schiff (PAS). Immunohistochemical staining was performed using an anti-nuclear factor kappa-B (NF-*κ*B) p65 primary antibody (Santa Cruz Biotechnology, Dallas, TX, USA), followed by incubation with horseradish peroxidase-conjugated anti-rabbit IgG secondary antibody (Santa Cruz Biotechnology). Images were captured using an Olympus BX53 microscope (Tokyo, Japan). Tubular damage in PAS-stained kidney sections was scored according to the level of cortical tubular injury as previously described: 0, normal; 1, 1–10%; 2, 11–25%; 3, 26–45%; 4, 46–75%; and 5, 76–100% [12]. The number of NF-*κ*B-activated cells in each section was determined by counting the number of cells showing nuclear p65-positive staining in 10 fields per slide (×200).

### 2.4. Terminal Deoxynucleotidyl Transferase-Mediated dUTP Nick End Labeling (TUNEL) Assay

Apoptotic cell death was examined in the kidney sections using in situ cell death detection kit (Roche, Mannheim, Germany), according to the manufacturer's instructions. Briefly, the kidney sections were deparaffinized in xylene, rehydrated using descending grades of ethanol, and permeabilized for 30 min at room temperature with proteinase K (20 *μ*g/mL, Sigma-Aldrich) in 10 mM Tris-HCl, pH 7.4–8. After washing with phosphate-buffered saline (PBS), kidney sections were incubated in the TUNEL reaction mixture for 1 h at 37°C. Images were captured using an Olympus CKX41 inverted microscope. The number of TUNEL-positive cells was counted in 5 fields per slide (×200).

### 2.5. Western Blot Analysis

Kidney tissues were prepared using a lysis buffer (20 mM Tris-HCl (pH 7.4), 1% Nonidet P-40 (NP-40), 5 mM ethylenediaminetetraacetic acid (EDTA), 2 mM Na_3_VO_4_, 100 mM NaF, 10 mM Na_4_P_2_O_7_, 100 *μ*M phenylmethylsulfonyl fluoride (PMSF), 7 *μ*g/mL aprotinin, and 7 *μ*g/mL leupeptin). Proteins were resolved by sodium dodecyl sulfate- (SDS-) polyacrylamide gel electrophoresis and then transferred to polyvinylidene difluoride (PVDF) membrane. After blocking, the membrane was incubated with the following primary antibodies: anti-Bax (Cell Signaling Technology, Danvers, MA, USA), anti-caspase-3 (Cell Signaling Technology), anti-poly(ADP-ribose) polymerase-1 (PARP-1; Cell Signaling Technology), anti-heme oxygenase-1 (HO-1; Santa Cruz Biotechnology), anti-NAD(P)H:quinone oxidoreductase 1 (NQO1; Santa Cruz Biotechnology), and anti-*β*-actin (Sigma-Aldrich). The membrane was washed and incubated with a horseradish peroxidase-conjugated secondary antibody, and signals were detected using an enhanced chemiluminescence (ECL) Western blotting detection system (ImageQuant LAS4000; GE Healthcare Life Sciences, Pittsburgh, PA, USA). Relative protein expression was quantified using NIH ImageJ software.

### 2.6. Statistical Analysis

Data are expressed as the mean ± standard error of the mean (SEM). The differences between groups were analyzed using one-way analysis of variance (ANOVA) followed by Bonferroni's post hoc test. A *P* value < 0.05 was considered statistically significant.

## 3. Results

### 3.1. Gemigliptin Attenuated Renal Dysfunction and Tubular Damage in Cisplatin-Treated Mice

**Figure 1:**
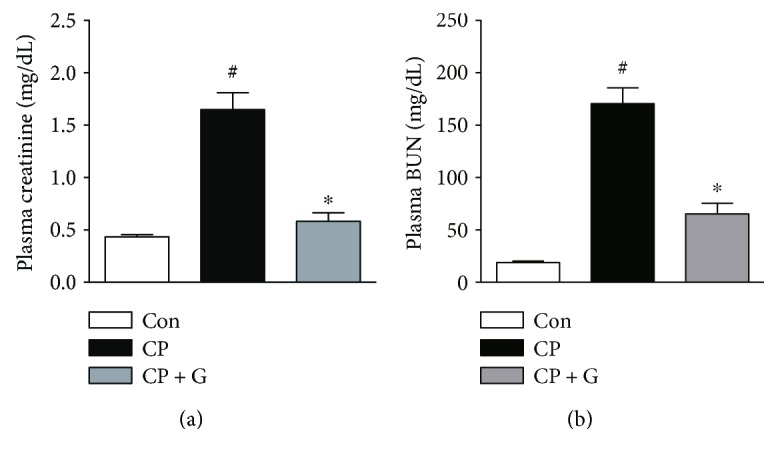
Effects of gemigliptin pretreatment on renal function in cisplatin-treated mice. Plasma levels of creatinine (a) and BUN (b). Con: control, *n* = 6; CP: cisplatin, *n* = 6; and CP + G: cisplatin + gemigliptin, *n* = 6. All data are expressed as the mean ± SEM. ^#^*P* < 0.01 versus Con and ^∗^*P* < 0.01 versus CP.

**Figure 2:**
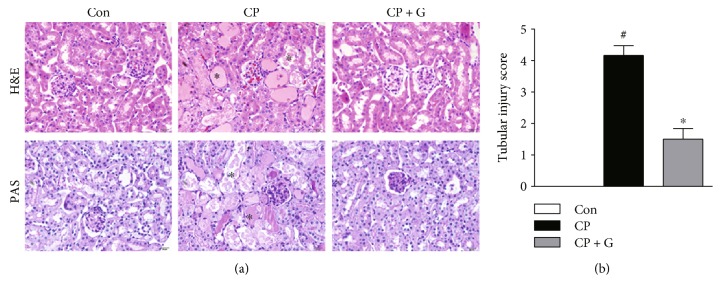
Effects of gemigliptin pretreatment on renal histology in cisplatin-treated mice. (a) Representative images of hematoxylin and eosin (H&E, ×400) and periodic acid-Schiff (PAS, ×400) staining of kidney sections. Asterisks indicate tubule damage. (b) Tubular injury score. Con: control, *n* = 6; CP: cisplatin, *n* = 6; and CP + G: cisplatin + gemigliptin, *n* = 6. All data are expressed as the mean ± SEM. ^#^*P* < 0.01 versus Con and ^∗^*P* < 0.01 versus CP.

Mice were intraperitoneally injected with cisplatin at 20 mg/kg to induce acute kidney injury. Mice treated with cisplatin alone showed a marked deterioration of renal function, as evidenced by elevated plasma levels of creatinine (Figure 1(a)) and BUN (Figure 1(b)) 72 h after cisplatin treatment. Interestingly, pretreatment with gemigliptin significantly attenuated cisplatin-induced elevation of plasma creatinine and BUN levels, compared to that in mice treated with cisplatin alone. H&E and PAS staining revealed that cisplatin-treated mice exhibited severe renal histological abnormalities, including tubular cell death, tubular dilatation, and tubular cast formation (Figures 2(a) and 2(b)). Remarkably, these tubular abnormalities were significantly ameliorated in gemigliptin-pretreated mice.

**Figure 3:**
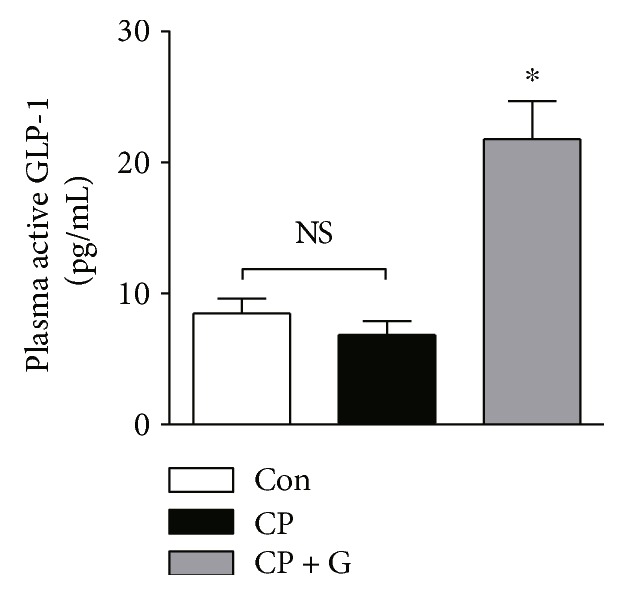
Effects of gemigliptin pretreatment on plasma levels of active GLP-1. Con: control, *n* = 6; CP: cisplatin, *n* = 6; and CP + G: cisplatin + gemigliptin, *n* = 6. All data are expressed as the mean ± SEM. ^∗^*P* < 0.01 versus CP. NS: not significant.

Given that DPP-4 inhibitors enhance endogenous GLP-1 levels, we measured the plasma levels of GLP-1 in all experimental groups. Expectedly, plasma GLP-1 levels were significantly higher in the gemigliptin-pretreated mice than in cisplatin alone-treated mice at the end of the study (Figure 3). Taken together, these results suggest that pretreatment with gemigliptin attenuates cisplatin-induced acute kidney injury, and this effect is possibly related to the elevation of active GLP-1 levels.

### 3.2. Gemigliptin Attenuated Cisplatin-Induced Apoptotic Cell Death

**Figure 4:**
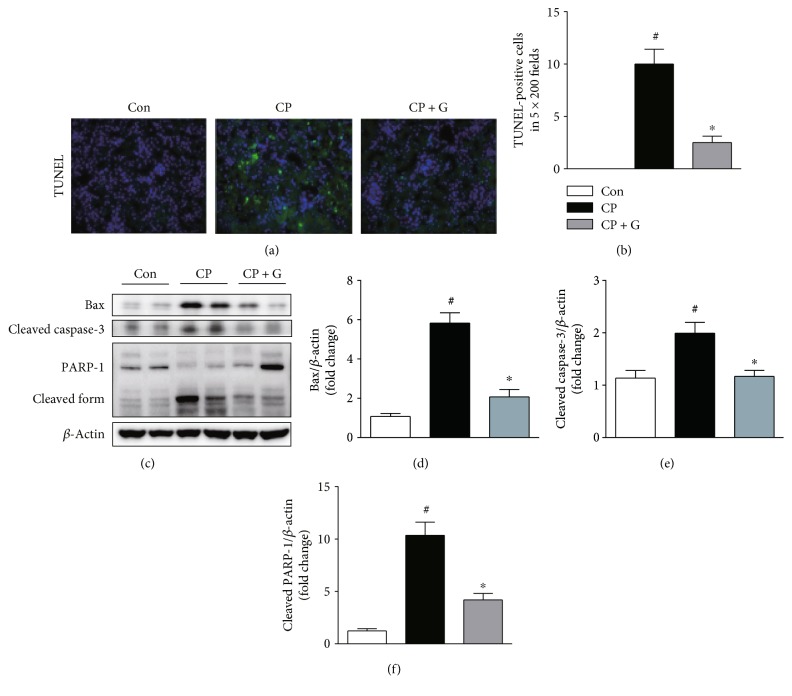
Effects of gemigliptin pretreatment on cisplatin-induced apoptotic cell death in the kidneys. (a) TUNEL staining (×200). (b) The number of TUNEL-positive cells in 5 fields (×200). (c) Western blot analysis of the expression of Bax, cleaved caspase-3, cleaved PARP-1, and *β*-actin in the kidneys. The graphs showing the results of quantitative analysis of Bax (d), cleaved caspase-3 (e), and cleaved PARP-1 (f) among groups. Con: control, *n* = 6; CP: cisplatin, *n* = 6; and CP + G: cisplatin + gemigliptin, *n* = 6. All data are expressed as the mean ± SEM. ^#^*P* < 0.01 versus Con and ^∗^*P* < 0.01 versus CP.

Apoptotic death of renal tubular cells plays a central role in the pathogenesis of cisplatin-induced nephrotoxicity [2]. To evaluate the mechanisms underlying the protective effects of gemigliptin against cisplatin-induced nephrotoxicity, we performed TUNEL staining of kidney sections obtained from each experimental group. The number of TUNEL-positive tubular cells in the kidneys of gemigliptin-pretreated mice was markedly lower than that in the kidneys of mice treated with cisplatin alone (Figures 4(a) and 4(b)). Cisplatin-induced increase in the level of the proapoptotic protein, Bax, in the kidneys was significantly reduced by pretreatment with gemigliptin (Figures 4(c) and 4(d)). Moreover, pretreatment with gemigliptin significantly decreased the levels of cleaved caspase-3 and cleaved PARP-1 in the kidneys of cisplatin-treated mice (Figures 4(c), 4(e), and 4(f)). These results show that pretreatment with gemigliptin attenuates cisplatin-induced apoptotic cell death in the renal tubules of mice.

### 3.3. Gemigliptin Attenuated Cisplatin-Induced Inflammatory Responses

**Figure 5:**
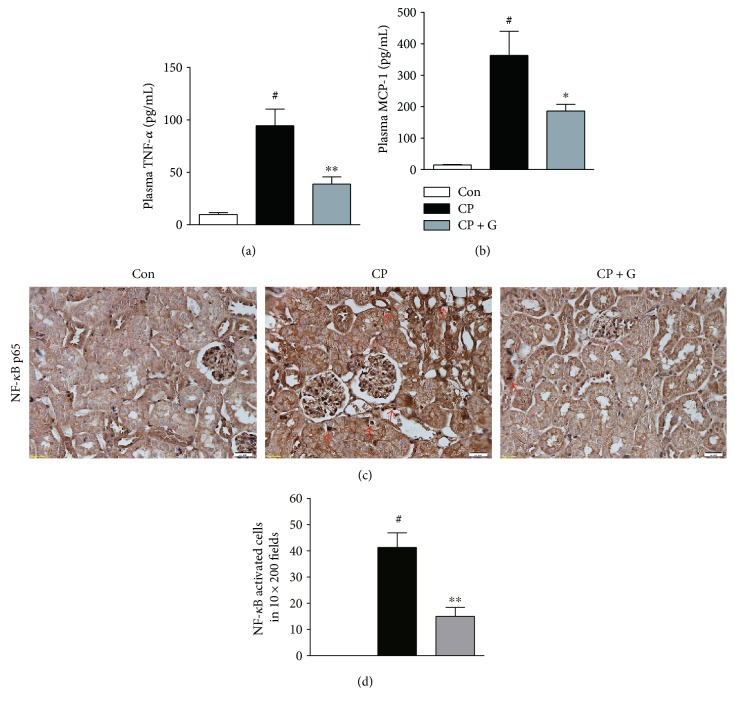
Effects of gemigliptin pretreatment on cisplatin-induced inflammatory responses. Plasma levels of TNF-*α* (a) and MCP-1 (b). (c) Immunohistochemical staining of nuclear NF-*κ*B p65 in the kidneys (×200). Red arrows indicate cells showing nuclear p65-positive staining. (d) The number of NF-*κ*B-activated cells in 10 fields (×200). Con: control, *n* = 6; CP: cisplatin, *n* = 6; and CP + G: cisplatin + gemigliptin, *n* = 6. All data are expressed as the mean ± SEM. ^#^*P* < 0.01 versus Con, ^∗^*P* < 0.05 versus CP, and ^∗∗^*P* < 0.01 versus CP.

Besides direct cellular toxicity, inflammation has been considered an important pathogenic factor in cisplatin-induced nephrotoxicity [4]. Among various mediators of inflammatory renal injury, TNF-*α* is the prototypical inflammatory cytokine, which plays a central role in cisplatin-induced nephrotoxicity [13]. Thus, we next examined the effect of gemigliptin on plasma levels of TNF-*α*. Pretreatment with gemigliptin significantly decreased TNF-*α* plasma levels in cisplatin-treated mice (Figure 5(a)). Similarly, the plasma levels of MCP-1, a key chemokine that regulates the migration and infiltration of monocytes/macrophages, in gemigliptin-pretreated mice were significantly lower than those in cisplatin alone-treated mice (Figure 5(b)).

NF-*κ*B is a well-known transcriptional regulator of cytokine/chemokine gene expression. Therefore, we next performed an immunohistochemical analysis to evaluate the nuclear translocation of NF-*κ*B p65 subunit in the kidney sections. Mice treated with cisplatin alone exhibited increased nuclear staining of p65 in the renal tubular cells (Figures 5(c) and 5(d)). Pretreatment with gemigliptin significantly reduced nuclear staining of p65 in the kidneys of cisplatin-treated mice. Taken together, these results suggest that pretreatment with gemigliptin attenuates cisplatin-induced inflammatory responses.

### 3.4. Gemigliptin Increased the Expression of HO-1 and NQO1 in the Kidneys of Cisplatin-Treated Mice

**Figure 6:**
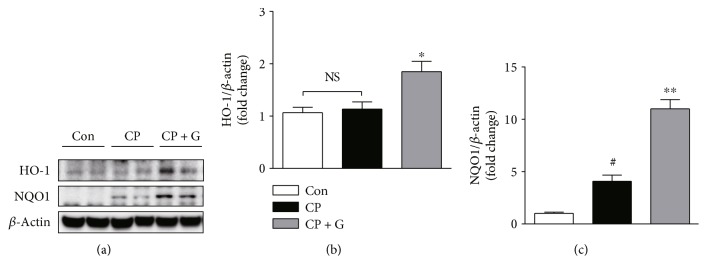
Effects of gemigliptin pretreatment on protein expression of HO-1 and NQO1 in the kidneys. (a) Western blot analysis of the expression of HO-1, NQO1, and *β*-actin in the kidneys. The graphs showing the results of quantitative analysis of HO-1 (b) and NQO1 (c) among groups. Con: control, *n* = 6; CP: cisplatin, *n* = 6; and CP + G: cisplatin + gemigliptin, *n* = 6. All data are expressed as the mean ± SEM. ^#^*P* < 0.01 versus Con, ^∗^*P* < 0.05 versus CP, and ^∗∗^*P* < 0.01 versus CP. NS: not significant.

Nrf2 is a key transcription factor that regulates the expression of several cytoprotective genes, such as HO-1 and NQO1, and protects the kidneys against various acute insults [14]. Recent studies have reported that sitagliptin, a prototype DPP-4 inhibitor, upregulates the expression of HO-1 and NQO1 in rat kidneys [15, 16]. In addition, accumulating evidence suggests that HO-1 and NQO1 play a protective role against cisplatin-induced nephrotoxicity [17, 18]. Thus, we next examined the effect of gemigliptin pretreatment on the expression of HO-1 and NQO1 in the kidneys of cisplatin-treated mice. Western blot analysis showed that pretreatment with gemigliptin significantly increased the expression of HO-1 (1.6-fold) and NQO1 (2.7-fold) in the kidneys of cisplatin-treated mice (Figures 6(a)–6(c)). These results suggest that induction of HO-1 and NQO1 expression by gemigliptin possibly plays a central role in the protection against cisplatin-induced nephrotoxicity.

## 4. Discussion

DPP-4 inhibitors are widely used antihyperglycemic agents, which act to promote pancreatic *β*-cell function via prevention of the inactivation of GLP-1. Recently, the pleiotropic extrapancreatic actions of DPP-4 inhibitors have drawn much attention owing to their potential applications in various diseases [5–8]. Indeed, recent studies have highlighted the potential beneficial effects of DPP-4 inhibitors in chronic kidney disease [8]. In addition, DPP-4 inhibitors ameliorated ischemia-reperfusion-induced acute kidney injury [19]. However, the effects of DPP-4 inhibitors against cisplatin-induced nephrotoxicity and the underlying mechanisms have not been fully elucidated yet. In the present study, we showed that gemigliptin, a newly developed DPP-4 inhibitor, protected against cisplatin-induced nephrotoxicity, possibly via inhibition of apoptotic death of renal tubular cells and inflammatory responses. In addition, elevated plasma levels of active GLP-1 were observed in gemigliptin-pretreated mice after cisplatin treatment, compared to that in the mice treated with cisplatin alone. A previous study showed that treatment with a GLP-1 receptor agonist or alogliptin, another DPP-4 inhibitor, attenuated cisplatin-induced acute kidney injury, and the knockdown of renal GLP-1 receptor expression *in vivo* by small interfering RNA reversed the renoprotective effects of alogliptin [20]. Thus, our findings suggest that gemigliptin-induced increase in GLP-1 levels possibly contributes to its protective effects against cisplatin-induced nephrotoxicity.

The mechanisms underlying cisplatin-induced nephrotoxicity are complex and involve multiple pathways. Among them, apoptosis of renal tubular cells has emerged as a central pathogenic process in cisplatin-induced nephrotoxicity [2–4]. Cisplatin can activate the proapoptotic Bcl-2 family proteins, such as Bax, which induces porous defects in the outer membrane of the mitochondria, resulting in the release of cytochrome c to the cytosol. Then, cytochrome c promotes the assembly of the apoptosome that results in caspase-9 activation and subsequent activation of the executioner caspases, including caspase-3. In the present study, we found that the number of apoptotic tubular cells detected by TUNEL staining in the kidneys of gemigliptin-pretreated mice was markedly lower than that in the mice treated with cisplatin alone. In addition, pretreatment with gemigliptin significantly reduced the expression of Bax and cleaved caspase-3 in the kidneys of cisplatin-treated mice. Moreover, the expression of the cleaved form of PARP-1, a typical caspase-3 substrate, in the kidneys of cisplatin-treated mice was markedly reduced by gemigliptin pretreatment. These results reveal the protective actions of gemigliptin against apoptotic cell death of the renal tubular cells in cisplatin-induced renal injury. In agreement with our findings, previous studies have shown that DPP-4 inhibitors have potent antiapoptotic effects in various types of cells [10, 11, 21–24]. In addition, DPP-4 inhibitors have been shown to protect against kidney damage induced by nephrotoxic insults, largely via inhibition of apoptosis [20, 25–27].

Besides the direct cellular toxicity, inflammatory responses contribute to the development and progression of renal injury during cisplatin treatment [2–4]. Previous studies have shown that cisplatin induces nuclear translocation of NF-*κ*B, which promotes the expression of proinflammatory mediators [28, 29]. In the present study, immunohistochemical detection of NF-*κ*B p65 subunit showed that cisplatin-induced increase in nuclear staining of p65 in the renal tubular cells was significantly reduced by gemigliptin pretreatment, which suggests that inhibition of NF-*κ*B activity by gemigliptin may be responsible for its suppressive effects against cisplatin-induced inflammation. Among numerous proinflammatory cytokines and chemokines under the control of NF-*κ*B, TNF-*α* is recognized as a key regulator in cisplatin-induced inflammatory responses. A previous study showed that inhibitors of TNF-*α* production and monoclonal antibodies against TNF-*α* reduced the serum and renal TNF-*α* protein levels and attenuated cisplatin-induced increase in the expression of other proinflammatory mediators, including MCP-1, which resulted in the amelioration of cisplatin-induced nephrotoxicity [13]. In addition, TNF-*α*-knockout mice were resistant to cisplatin-induced nephrotoxicity. Subsequent studies have shown that TNF-*α* plays a critical role in the induction of proinflammatory mediators and recruitment of inflammatory cells during cisplatin-induced nephrotoxicity [30, 31]. In the present study, we found that pretreatment with gemigliptin significantly decreased the plasma levels of TNF-*α* and MCP-1 in cisplatin-treated mice. Consistent with our findings, a recent study showed that sitagliptin ameliorated NF-*κ*B activation and TNF-*α* production in doxorubicin-induced cardiotoxicity [23]. Moreover, accumulating evidence suggests that DPP-4 inhibitors exert anti-inflammatory effects in various organs, including the brain [21], liver [22], heart [24], and kidneys [25–27].

The transcription factor Nrf2 plays a critical role in regulating the susceptibility of multiple organs, including the kidneys, to various chemical insults [14]. Cisplatin-induced nephrotoxicity was exacerbated in Nrf2-knockout mice [32], whereas pretreatment with Nrf2 inducers prevented the complications of cisplatin [32, 33]. In addition, accumulating evidence showed that HO-1 and NQO1, two important target genes of Nrf2, play a central role in the protection against cisplatin-induced nephrotoxicity [17, 18]. HO-1 is an inducible enzyme that catalyzes the oxidative degradation of heme to form biliverdin, carbon monoxide, and free iron [17]. These end products exert potent antioxidant, antiapoptotic, and anti-inflammatory actions. Genetic deletion of HO-1 aggravated the structural and functional damage during cisplatin-induced nephrotoxicity [34–36], whereas overexpression of HO-1 was protective [36], which suggested a protective role of HO-1 in cisplatin-induced nephrotoxicity. Moreover, treatment with hemin, an inducer of HO-1, alleviated cisplatin-induced nephrotoxicity [34]. NQO1, another important target gene of Nrf2, is also an inducible enzyme that regulates the intracellular redox status and has cytoprotective effects against various insults [18]. Recently, several studies have shown that the pharmacological activation of NQO1 attenuates cisplatin-induced nephrotoxicity [12, 37]. In the present study, we found that pretreatment with gemigliptin increased the expression of HO-1 and NQO1 in the kidneys of cisplatin-treated mice. Consistent with our findings, recent studies reported that sitagliptin upregulated HO-1 and NQO1 expression in rat kidneys, which protected against acute ischemia-reperfusion injury [15, 16]. Although the exact molecular mechanisms underlying the protective effects of gemigliptin against cisplatin-induced nephrotoxicity need to be elucidated in further studies, our results provide evidence that the induction of HO-1 and NQO1 expression by gemigliptin possibly plays a central role in the protection against cisplatin-induced nephrotoxicity.

In conclusion, the present study suggests that DPP-4 inhibition by gemigliptin protects against cisplatin-induced nephrotoxicity through inhibition of renal tubular cell apoptotic death and inflammatory responses via elevation of plasma active GLP-1 levels. In addition, induction of HO-1 and NQO1 by gemigliptin is probably involved in the protection against cisplatin-induced nephrotoxicity. Fortunately, a clinical trial examining whether gemigliptin can protect against cisplatin-induced nephrotoxicity is ongoing in the Republic of Korea [38]. Hopefully, these efforts will lead to the development of a promising new protective strategy against cisplatin-induced nephrotoxicity.
